# Natural course of refractive errors in early onset inherited retinal diseases

**DOI:** 10.1038/s41433-025-03987-9

**Published:** 2025-09-12

**Authors:** Rotem Azmon, Ben Ezra Kahtan, Karen Hendler, Claudia Yahalom

**Affiliations:** 1 Faculty of Medicine, Hebrew University of Jerusalem, Jerusalem, Israel; 2 Department of Ophthalmology, Hadassah Medical Center, Jerusalem, Israel

**Keywords:** Diseases, Signs and symptoms

## Abstract

**Background:**

Inherited retinal diseases (IRDs) are a leading cause of visual impairment in children and young adults. Individuals with IRDs have an increased prevalence of high refractive errors (REs). This study aims to characterise the natural progression of REs in patients with early onset IRDs and identify associations with specific IRDs and genes.

**Methods:**

Retrospective cohort study of patients diagnosed with IRD’s up to the age of 10 years. Data collected included demographic information, IRD type, molecular analysis (when available), and cycloplegic REs from the first and last visits.

**Results:**

A total of 199 patients (384 refractive measurements) were included in this study. Retinitis Pigmentosa (RP) and Achromatopsia were associated with high hypermetropia in early visits, with a decreasing RE trend over time. *CNGA3, CNGB3*, and *CRB1* were associated with high hypermetropia, remaining high with time in *CRB1*. In contrast, Congenital Stationary Night Blindness (CSNB) and Blue Cone Monochromacy (BCM) demonstrated high myopia, worsening over time in CSNB, with an increasing rate in high myopia from 51.5% to 69.7% from first to last visit. Mean myopic progression in *TRPM1*-patients was 0.56 dioptres/year.

**Conclusions:**

In patients with early onset IRDs, refractive errors have a general tendency towards lower spherical equivalents with time. *TRPM1*-related myopia keeps progressing during the first decade of life, warranting regular screening and consideration of early myopia control interventions to mitigate the risk of myopia-related sight-threatening complications. High hypermetropia is common in RP, staying especially high in *CRB1*-related cases, highlighting the importance of early screening and refractive correction.

## Introduction

Inherited retinal diseases (IRDs) encompass a diverse group of genetic disorders characterised by retinal degeneration and loss of vision due to dysfunction or the premature death of retinal cells [1]. These conditions are among the leading causes of visual impairment and blindness in children and young adults, representing the second most common reason for low-vision certification amongst children and the primary cause among the working age population, accounting for 23% of cases [2]. IRDs comprise a wide range of clinically and genetically heterogeneous disorders, with prevalence varying geographically approximately from one in 500 to one in 3000 [3].

High refractive errors (REs), such as myopia and hypermetropia, are common ocular disorders and major contributors to global vision impairment [4]. High myopia, in particular, can significantly affect vision due to complications such as retinal detachment, progressive choroidal degeneration, and glaucoma [5]. This issue is of growing concern as studies predict a global increase in myopia and high myopia prevalence, with myopia expected to affect 50% of the world population by 2050 [6].

It is well established that patients with IRDs often exhibit high ametropia [7]. However, there is limited data on the specific tendencies of different IRD subtypes toward particular RE patterns. Generally, IRDs are associated with myopic refractive errors, particularly in X-linked congenital stationary night blindness (CSNB), X-linked retinitis pigmentosa (RP), cone dystrophies, and some cone-rod dystrophies [7–10]. Conversely, hypermetropia is commonly observed in Leber’s congenital amaurosis (LCA) [11].

With significant advancements in molecular diagnostics, the ability to identify specific causative genes for IRDs has improved dramatically over the past decade. However, few studies have investigated the potential link between specific IRD-associated genes and RE patterns [12, 13].

This study aims to characterise the refractive natural history of REs across different IRD subtypes in children.

## Methods

This retrospective cohort study was conducted at a low vision clinic within a tertiary hospital, which serves as a national referral centre for patients with visual impairment. The clinic operates with a multidisciplinary team, including ophthalmologists, low vision optometrists, a social worker, and genetic counsellors. The study was approved by the hospital’s institutional ethics committee.

Patients diagnosed with an IRD who were followed at our clinic up to the age of 10 years between January 2018 and March 2024 were included in the study. The IRD diagnosis was based on typical clinical signs, electrophysiology (ERG), and/or molecular testing, when available. Data were collected from medical records, including gender, age, IRD type, follow-up duration, cycloplegic refractive error (RE), and genotype when available. Exclusion criteria included patients who had any myopia control treatment.

For refractive status analysis, we used spherical equivalent (SE) as per standard practice, calculated as: SE = Sphere + (Astigmatism / 2). RE were classified into seven sub-groups, based in SE values and included mild (≤−1.5 to <−3), moderate (≤−3 to < −6) to high (≤ −6) myopia; emmetropia ( + 1.5 to <−1.5); mild (≤ +1.5 to < +3), moderate (≤+3 to <+6) and high (≥ +6) hypermetropia, consistent with previous publications [13].

### Statistical analysis

Statistical analysis was carried out using the IBM SPSS Statistics program, version 29. All tests applied were two-tailed, and a p-value of 0.05 or less was considered statistically significant.

To test the association between two categorical variables, the Chi-square or Fisher’s exact test were used. The comparison of a quantitative variable between three independent groups or more was performed by using the Kruskal-Wallis non-parametric test, and for pair-wise comparison two independent groups by using the Mann-Whitney (M-W) non-parametric test. The M-W test was with the Bonferroni correction of the significance level. The Spearman non-parametric correlation coefficient was calculated for assessing the strength of the association between two quantitative variables. Testing the change in a quantitative variable was performed by using the Wilcoxon, signed, paired ranks test. Non-parametric tests were applied due to the small sample sizes and the non-normal distribution of some of the variables analysed.

## Results

From an initial cohort of 199 patients, 185 had at least two visits. A total of 384 refractive measurements were analysed. The mean age at diagnosis was 3.6 years (SD = 2.8; median: 2.7; range: 0.2–10). The mean follow-up interval between the first and last refraction was 5.6 years (SD = 3.1). Gender distribution showed that 116 (58.3%) were male and 83 (41.7%) were female.

A strong positive correlation in spherical equivalent (SE) was observed between the left eye (LE) and right eye (RE) for each subject, both at the first visit (*r* = 0.982, *p* < 0.01) and the last visit (*r* = 0.980, *p* < 0.01). Given this high correlation, the right eye was used as a representative indicator for both eyes in the analysis.

**Fig. 1:**
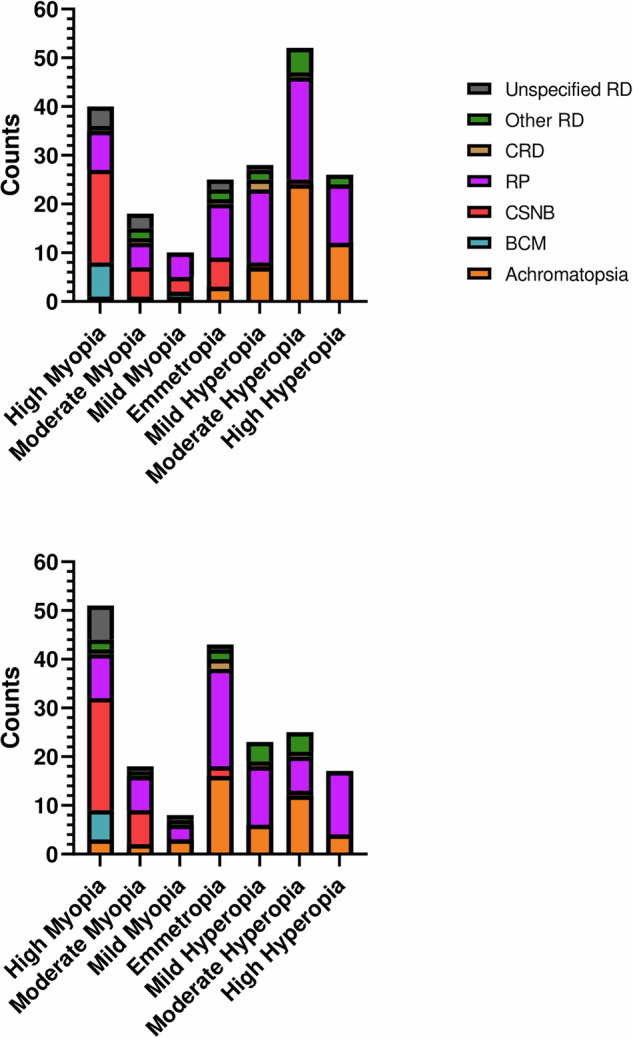
Distribution of studied patients with IRDs across refractive error groups. The upper graph represents the first visit, while lower graph depicts the last visit. LCA patients (N:22) are included among RP patients.

The most common IRDs in our cohort were RP (N:55), followed by achromatopsia (N:48), CSNB (N:36) and LCA (N:22), together comprising 80.9% of the entire cohort. The distribution of different RE for the first visit is showed in Fig. 1.

The mean SE at the first visit was around 0 D (median 1.9 D) and for the last visit mean SE was −1.7 D (median −0.4 D), with a high standard deviation of 6 D and 6.1 D respectively, suggesting high heterogenicity within the IRDs groups. Regarding astigmatism, it remained rather stable with values of 1.6 and 2.1 (first and last visits respectively) and did not show any specific correlation with any subtypes of IRDs or genes studied.

When conducting a categorical analysis of RE among all studied patients, we observed that both moderate and high myopia increased by the last visit, with patients swapping between different refractive categories towards the myopic end (14 new high myopic children by last visit). Full details regarding refractive categories changes with time can be found at Table 1.

**Table 1:** Spherical Equivalent (SE) Categorical Distribution Change Between Visits.

Category	First visit *N* (%)	Last visit *N* (%)	Number of patients that changed refractive category (%)*
High myopia	40 (20.1)	51 (27.6)	+14 (7.6)
Moderate myopia	18 (9)	18 (9.7)	+2 (1.1)
Mild myopia	10 (5)	8 (4.3)	−1 (0.5)
Emmetropia	25 (12.6)	43 (23.2)	+19 (10.3)
Mild Hypermetropia	28 (14.1)	23 (12.4)	−4 (2.2)
Moderate Hypermetropia	52 (26.1)	25 (13.5)	−25 (13.5)
High Hypermetropia	26 (13.1)	17 (9.2)	−5 (2.7)

^*^Net progression was calculated with relation only to the 185 patients who completed follow-up.

**Fig. 2:**
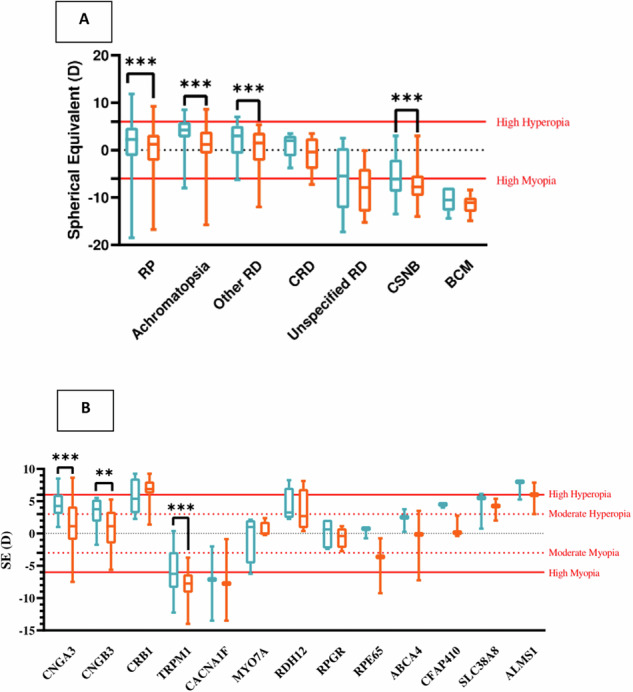
Spherical equivalence change during studied period. Spherical Equivalence values across specific IRD diagnosis (**A**) and genes (**B**). First visit in blue and last visit in orange. Statistically significant differences between visits are marked. Only patients who completed both visits were included. **- *p*-value < 0.01; ***-*p*-value < 0.001.

Blue Cone Monochromacy (BCM) and Congenital Stationary Night Blindness (CSNB) tended to present with myopia at the first visit, whereas patients with Retinitis Pigmentosa (RP) and Achromatopsia were initially hypermetropic, showing significant differences by pairwise comparisons (*p*-value < 0.001). All four groups exhibited progression toward lower SE values at their last visit. Decreasing SE values were significant in RP, achromatopsia, and CSNB (*p*-value ≤ 0.01) Fig. 2A.

Regarding categorical analysis, all patients with BCM were highly myopic and remained so throughout the study. Among patients with CSNB, 78.8% were myopic at the first visit (with 51.5% exhibiting high myopia). By the last visit, 90.9% were myopic (with 69.7% showing high myopia).

In patients with achromatopsia, 89.1% were hyperopic at the first visit (23.9% highly hyperopic). By the last visit, only 47.8% remained hyperopic.

Among patients with RP, 62.0% exhibited hyperopia (12.7% highly hyperopic) at the first visit, which decreased to 45.1% at the last visit Fig. 1.

### Molecular analysis

Genetic testing was performed on 162 patients (81.4%), revealing pathogenic variants in 137 individuals (84.6%). The most common genes identified were *CNGA3* and *TRPM1* (Table 2). Only genes identified in three or more patients across both visits were analysed, with distributions provided for each visit, mean age at exam, follow-up time, and RE. The spherical equivalent (SE) distribution varied according to the genes involved. There was a statistically significant relationship between genes and both SE and refractive categories distribution along both visits (*p*-value < 0.001). SE decreased significantly in *CNGA3* and *TRPM1* from the first to last visit (*p*-value < 0.001). Details about the different SE progression among studied genes can be found in Table 2.

**Table 2:** Spherical Equivalent (SE) Distribution Across Different Genes.

	First Visit			Last Visit			SE Annual progression		
Gene	*N* (%)	Age (SD)	SE (SD)	*N* (%)	Age (SD)	SE (SD)	Years (SD)	mean (SD)*	Median [Min, Max]
***CNGA3***	29 (21.2)	2.0 (2.1)	4.5 (1.9)	28 (21.5)	8.8 (3.6)	1.4 (4.0)	6.7 (3.6)	−0.4 (0.5)	−0.4 [−1.7, 0.4]
***TRPM1***	21 (15.3)	3.0 (2.4)	−5.5 (3.8)	21 (16.2)	8.6 (3.5)	−8.2 (2.7)	5.6 (3.0)	−0.6 (0.5)	−0.4 [−1.8, 0.1]
***CNGB3***	9 (6.6)	1.3 (1.5)	3.3 (2.4)	9 (6.9)	7.5 (4.2)	0.7 (3.4)	6.2 (4.1)	−0.6 (0.7)	−0.5 [−2.4, 0.1]
***CRB1***	8 (5.8)	2.2 (2.1)	5.7 (2.6)	8 (6.2)	8.1 (3.0)	6.6 (2.4)	5.9 (3.3)	0.1 (0.3)	0.2 [−0.3, 0.5]
***CACNA1F***	5 (3.7)	5.8 (2.9)	−7.4 (4.1)	3 (2.3)	9.9 (1.1)	−7.4 (6.3)	4.4 (3.5)	0.0 (0.1)	0.0 [−0.1, 0.1]
***RPGR/RGPIR***	5 (3.7)	7.3 (2.1)	0.1 (2.2)	5 (3.9)	11.5 (2.9)	−0.7 (1.6)	4.3 (2.8)	−0.3 (0.4)	−0.1 [−0.9, 0.1]
***ALMS1***	4 (2.9)	3.1 (3.8)	7.9 (2.1)	3 (2.3)	7.5 (2.6)	5.6 (2.5)	6.3 (2.6)	−0.2 (0.1)	−0.3 [−0.3, 0.0]
***MYO7A***	4 (2.9)	4.1 (4.0)	−0.5 (3.9)	4 (3.1)	8.1 (4.4)	0.5 (1.2)	4.0 (1.7)	0.3 (0.8)	0.1 [-0.4, 1.5]
***RDH12***	4 (2.9)	4.5 (2.7)	4.3 (2.7)	4 (3.1)	8.6 (3.5)	3.5 (3.3)	4.2 (1.6)	−0.1 (0.3)	−0.2 [−0.4, 0.2]
***ABCA4***	4 (2.9)	5.8 (4.9)	2.2 (1.8)	3 (2.3)	13.0 (3.1)	−1.3 (5.5)	7.2 (6.1)	−0.3 (0.3)	-0.2 [−0.7, 0.0]
***CFAP410***	4 (2.9)	2.3 (1.4)	5.8 (3.0)	3 (2.3)	8.0 (2.6)	0.8 (1.7)	5.6 (2.2)	−0.8 (0.5)	−1.0 [−1.0, 0.2]
***RPE65***	3 (2.2)	5.1 (4.0)	0.3 (0.9)	3 (2.3)	9.5 (5.8)	−4.5 (4.3)	4.4 (2.0)	−1.6 (2.0)	−0.8 [−3.9, −0.3]
***SLC38A8***	3 (2.2)	1.4 (1.3)	4.1 (2.9)	3 (2.3)	5.3 (1.7)	3.9 (1.7)	3.9 (0.5)	−0.1 (0.3)	−0.2 [−0.4, 0.3]

*N* number, *SE* spherical equivalent.

*Annual progression values were obtained using IBM SPSS Statistics program, based on mean individual patient’s progression.

Regarding the progression of SE within each group between visits, statistically significant progression was observed in *CNGA3, TRPM1* (*p*-value < 0.001) and *CNGB3* (*p*-value = 0.008) Fig. 2B.

**Fig. 3:**
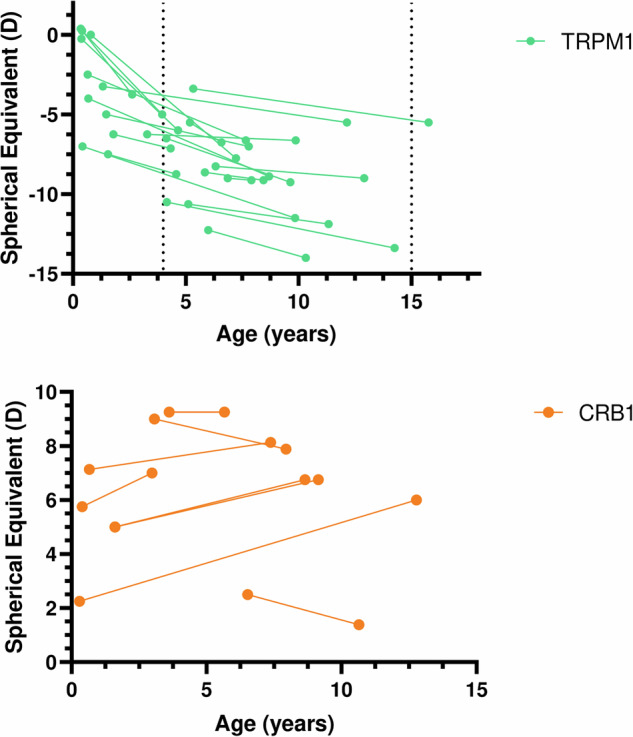
Longitudinal follow-up for studied patients with *TRMP1* and *CRB1* gene related IRDs according to age.

Longitudinal analysis of refractive error among most relevant studied genes revealed that in *TRMP1* patients with high myopia remained highly myopic, and 6 patients (60%) progressed from moderate to high myopia at the last visit reaching a total of 81.0% highly myopic patients. Mean myopic progression in *TRPM1*-patients was 0.56 dioptres/year (median 0.4) Table 2. Continuous myopic progression was observed in some patients during the first decade of life Fig. 3.

Regarding patients with *CRB1* mutations, most of them were hypermetropic (75% with high hypermetropia) at the first visit. At the last visit, 87.5% (7/8 patients) had high hypermetropia. Fig. 3. Annual progression in SE showed a mean of 0.1 D (median (0.2) Table 2.

*CFAP410* and *RPE65* genes showed a marked decrease in SE values; CFPAP410 showed a mean of myopic progression of -0.8 D (median -1 D), and *RPE65* showed a mean myopic progression of −1.6 D (median −0.8 D) during follow up Table 2.

## Discussion

This study highlights the distribution and evolution of refractive errors in patients with IRDs from an early age. Our results demonstrate a strong association between different IRDs and significant ametropia during the first decade of life, with high refractive errors present in approximately one-third of patients. Moreover, we observed considerable heterogeneity within IRDs, with distinct refractive error patterns correlating with specific diagnoses and genes.

To the best of our knowledge, our cohort represents one of the largest refractive error database in early onset IRDs to date, and the first study to examine RE longitudinal progression in patients with different types of IRDs from a young age over time.

In healthy paediatric populations, refractive error development and changes have been well-documented by Flitcroft and colleagues [14]. Their research suggests that between 3 months and 3.5 years of age, a series of key processes occur, including a shift in mean refraction from +2.00 D to approximately +0.75 D, a reduction in variability, and the emergence of a positively skewed refractive error distribution. These processes, collectively referred to as emmetropization, are most rapid during early childhood, continuing at a slower pace until around 6 years of age. This phenomenon is observed globally, even in populations with high myopia rates among children and adults.

Our findings support that emmetropization does not seem to occur in patients with IRDs from an early age. At the initial visit (mean age of 3.6 years), the mean refractive error was 0.00 D, median of 1.9 D with a high standard deviation of 6.0, accompanied by a notable prevalence of high hyperopia and myopia. After a mean follow-up of 5.6 years (mean age of 9 years), a significant myopic shift was observed, with a mean SE of −1.7 D, a median of −0.4 D and a persistently high SD of 6.1.

Our results align with previous studies, such as Hendriks and associates’ findings on CSNB patients, who demonstrated high myopia, especially in those with mutations in the *CACNA1F, NYX*, and *TRPM1* genes [12].

Similarly, other publications report that CSNB patients had a significant myopic shift over time [7]. Igelman and associates [15] focused on the progressive aspect of myopia among CSNB paediatric patients, analysing samples of 78 paediatric patients with CSNB, out of which 41 with *CACNA1F*, 22 with *NYX* and 15 with *TRPM1* genes. They concluded that all 3 genes showed myopic progression during follow-up with a mean annual progression for *TRPM1* patients of −0.326 D. Yassin later on [13], reported seven patients with *NYX* variants presenting with highly myopic values.

Poels recently published a study suggesting that the refractive error of children with CSNB changes minimally after the age of 4 years old [16]. In his study, only 12 patients with TRMP1-related CSNB, from whom only 9 had refractive follow up, limiting their conclusions regarding this gene specifically.

This study reinforced the association of *TRPM1* with high myopia, showing a more pronounced mean myopic progression of −0.56D per year and median of -0.41 D per year (as observed in our cohort of 21 patients). This myopic increase is seen all along the first decade of life, contrary to Poels observations in 2024 [16]. highlighting the need for regular monitoring and consideration of myopia control treatments. The high prevalence of *TRPM1*-related CSNB patients in our cohort is explained because of two known founder mutations that account for vast majority of cases in our population: a nonsense mutation c.880 A > T (p.Lys294*) and a large genomic deletion (36,445 bp) encompassing exons 2–7 of *TRPM1* [17].

In our cohort, patients with Blue Cone Monochromacy (BCM) also exhibited a strong association with high myopia. Our data, along with Semenov and colleagues’ study, supports the connection between BCM and high myopia [18]. Our cohort of 9 BCM patients had a mean SE of −8.6 D (median −7.9 D), and high myopia persisted over time with stable SE values.

Regarding *CRB1* gene, previous publications report significant hypermetropia [19–21] in coincidence with our results showing that all *CRB1* patients had high hypermetropia at the first visit, with a mean of +5.7 D (median 5.4 D). By the last visit, hypermetropia progressed to a mean of +6.6 D (median 6.9 D), with 87.5% of patients classified as highly hypermetropic. This trend was particularly prominent in patients with Leber Congenital Amaurosis (LCA) phenotype, emphasising the importance of early refractive correction.

Our study is one of the first to quantify the refractive progression in achromatopsia patients, showing a shift from mild hypermetropia toward emmetropization over time [12, 22].

While the findings regarding *RPGR* variants remain mixed, with some studies indicating a high myopia association, our cohort showed that *RPGR* patients were relatively emmetropic, supporting Yassin’s findings [12, 22]. Regarding *CFAP410* and *RPE65* genes, not much is known about their refractive natural history. In our cohort we saw a consistent lowering of their SE with time in both genes, but the number of patients was too small to get a reliable refractive pattern.

In conclusion, this study emphasises the critical need for early significant ametropia detection and tailored interventions to optimise visual outcomes in young patients with IRDs. Given the significant myopia progression in *TRMP1*-related CSNB, early treatment with of low-dose atropine or peripheral defocus glasses for slowing myopia progression, should be taken into consideration. In addition, in patients with *CRB1*-related LCA, early refractive monitoring is essential to avoid secondary amblyogenic effects of uncorrected high hypermetropia. These results underline the complexity of refractive outcomes in IRDs and highlight the need for further research to explore the interactions between genetic and environmental factors.

## Summary

### What was known before

- Significant refractive errors are common in inherited retinal diseases.
- CSNB is traditionally associated with high myopia.

### What this study adds

- TRMP1-related CSNB is associated to high myopia that keeps progressing during the first decade of life.
- CRB1-related early onset retinal dystrophies are associated with high hypermetropia that continues to increase with time.

## Data Availability

Data is available upon request.
